# 双通道三色谱柱气相色谱法测定成品车用汽油详细组成

**DOI:** 10.3724/SP.J.1123.2024.02013

**Published:** 2024-08-08

**Authors:** Changxiu LI, Yamin WANG, Yiwei ZHANG, Zheng WANG

**Affiliations:** 中石化石油化工科学研究院有限公司, 北京 100083; SINOPEC Research Institute of Petroleum Processing Co., Ltd., Beijing 100083, China

**Keywords:** 气相色谱, 含氧化合物, 苯胺类化合物, 车用汽油, 详细组成, 中心切割, gas chromatography (GC), oxygenate, anilines, motor gasoline, individual components, Deans switch

## Abstract

建立了一套双通道、三柱、三检测器的气相色谱分析系统,用于成品车用汽油的详细组成分析。采用双进样器同时进样方式将试样导入两个色谱进样口,第一个进样口(通道1)的试样中组分经非极性色谱柱(PONA柱)(50 m×0.20 mm×0.5 μm)分离,进入火焰离子化检测器(FID)检测;第二个进样口(通道2)的试样组分进入第二根PONA柱分离,依据事先设定的时间表,采用多次中心切割的方式将一些PONA柱上难分离组分的共流出峰切割至DM-624色谱柱(30 m×0.25 mm×1.4 μm)上进一步分离,经FID检测。这些难分离的组分包括与烃类共流出的醇醚类含氧化合物,一些汽油标准中禁止人为添加的非常规添加组分如甲缩醛、碳酸二甲酯、乙酸仲丁酯和苯胺类化合物,以及一些关键的难分离物质对如甲苯和2,3,3-三甲基戊烷。第二根PONA色谱柱通过中心切割出口连接一根阻尼柱与第三个检测器相连,以方便地确定合适的中心切割时间表。根据DM-624色谱柱上分离得到的难分离组分的峰面积和通道间的定量校正系数,推算出这些难分离组分在通道1的PONA色谱柱上的共流出峰中所占的色谱峰面积,从而实现共流出色谱峰的拆分。根据通道1的PONA柱上所有色谱峰的峰面积和定性结果,采用校正归一的方法可以得到车用汽油中含氧化合物、非常规添加组分和单体烃质量分数,并进一步计算得到样品的碳数族组成数据,消除了含氧化合物及非常规添加组分与烃类组分间的干扰问题。该方法对实际车用汽油的测定结果与标准方法GB/T 30519-2016、NB/SH/T 0663-2014及SH/T 0693-2000的测定结果有很好的一致性。对于添加有甲缩醛等某些非常规添加组分的样品,采用本方法也可以得到这些非常规添加组分的含量。通过本方法一次分析可以得到需要使用3~4个标准方法才能获得的分析数据,同时可以获得车用汽油的详细单体烃组成信息,方法具有较好的应用前景。

## Abstract (en)

A method based on a dual-channel gas chromatograph equipped with three columns and three detectors was established for the determination of individual components in finished motor gasoline. The gasoline samples were separately introduced into the two injection ports of the chromatograph using two autosamplers. The components of the sample introduced into the first injection port (channel 1) were separated on a nonpolar PONA column (50 m×0.20 mm×0.5 μm) for gasoline analysis and detected by an flame ionization detector (FID). The components of the sample introduced into the second injection port (channel 2) were separated on another PONA column. Oxygenates (e.g., ethers and alcohols), other unconventional and prohibited additives that would co-elute with the hydrocarbons (e.g., methylal, dimethyl carbonate, *sec*-butyl acetate, and anilines), and some difficult-to-separate hydrocarbon pairs (e.g., 2,3,3-trimethylpentane and toluene) eluted from the PONA column and entered a DM-624 column (30 m×0.25 mm×1.4 μm) to achieve further separation according to the switch timetable using the Deans switch procedure and detected by an FID. The peak of 3-methylpentane, a common component in gasoline samples, also entered the DM-624 column by the Deans switch procedure for calculation purposes. The peak areas of target components on the PONA column in channel 1 were calculated using the peak areas on the DM-624 column as well as those of 3-methylpentane on both the DM-624 and PONA columns in channel 1 with a calibration factor between the two channels. The peak areas of co-eluted components were obtained by subtracting the calculated peak areas of the target components from those of the co-eluted peaks. The mass percentages of the individual components were calculated according to the normalization method using all peak areas on the PONA column in channel 1 with relative response factors. The mass percentages of the oxygenates, anilines, and individual hydrocarbons were determined, and the group-type distribution was calculated according to the carbon number. Separation and quantitation interferences between the additives and hydrocarbons were eliminated using this procedure. Twenty oxygenates and unconventional additives, each with a mass percentage of approximately 3%, were added to a real motor gasoline-92 sample and analyzed using the proposed method. The recoveries of the target components were between 90.1% and 118.2% with relative standard deviations (RSDs) between 0.2% and 5.1% (*n*=6). The analysis of a real ethanol-gasoline sample showed that the RSDs of contents of most components was less than 3% (*n*=6). Because the heart-cut of peaks using Deans switch technique requires the precise repeatability of retention times, the retention-time repeatability of components on the PONA column in channel 2 was investigated over an extended period of time after thousands of runs of real-sample analysis. The retention times of the same component in several randomly selected motor gasoline-92 samples varied from 0.01 to 0.03 min, indicating that the proper timetable for the Deans switch remained stable for two years. The precise repeatability of retention times was achieved owing to the high precision of the electric pneumatic control of the chromatograph and the stability of the column used. Real finished motor gasoline samples with different octane numbers (gasoline-92, gasoline-95, and ethanol gasoline-95) were analyzed using the developed method, and the results acquired were consistent with those of standard methods (GB/T 30519-2016, NB/SH/T 0663-2014, and SH/T 0693-2000). If some unconventional additives (such as methylal) were added to gasoline samples, the contents of these unconventional additives could also be detected, which means one run of the proposed method could provide results corresponding to three or four runs of different standard methods. The acquisition of information on the individual components of finished motor gasoline will assist in research on precise gasoline blending.

为满足汽油排放指标要求,国家油品质量不断升级。2023年1月1日起全国开始实行国ⅥB汽油标准,与国Ⅴ汽油相比,该标准进一步降低了车用汽油中芳烃和烯烃的含量。成品车用汽油是由不同类型的调合组分汽油以一定的比例调合而成。为满足新的汽油标准,调合组分汽油和调合方案变得更加复杂多样,除基础的催化裂化(FCC)汽油、重整汽油外,还要更多地调入烷基化汽油、异构化汽油等组分汽油以及甲基叔丁基醚(MTBE)等含氧化合物,用以在降低芳烃和烯烃含量的同时提高汽油的辛烷值。同时,市场上也曾发现个别车用汽油中调入了车用汽油标准禁止人为添加的甲缩醛、乙酸仲丁酯、苯胺类化合物等非常规添加组分,这些组分会对车用汽油的使用性能造成影响,如甲缩醛价格低廉,可大大降低油品成本,但其溶解性强,会产生汽车橡胶密封圈发胀、溶解等不良影响;乙酸仲丁酯(SBA)和苯胺类化合物尽管可以在一定程度上提高汽油的辛烷值,但也会对油品性能带来不确定性或不利影响,如苯胺类化合物会影响汽油的氧化安定性。因此,获知调合组分汽油及成品车用汽油分子水平的组成信息,将为车用汽油的精准调合、品牌汽油生产及汽油质量控制提供支持。但是,由于调合组分汽油组成的复杂性,同时添加的含氧化合物和可能存在的非常规添加组分与烃类组分间的相互干扰,使得采用常规方法较难获得车用汽油准确的单体烃组成。

从目前的分析手段来看,车用汽油组成的测定在国Ⅵ汽油产品标准中采用GB/T 30519-2016方法^[1]^,该方法可以测定车用汽油的饱和烃、烯烃和芳烃的总量,但无法获得单个组分的含量,同时当车用汽油中添加有C_1_~C_4_醇、MTBE、乙基叔丁基醚(ETBE)、二异丙醚(DIPE)、叔戊基甲基醚(TAME)等含氧化合物或某些非常规添加组分时,必须通过其他的标准方法^[2,3]^进行修正。国Ⅵ汽油标准中同时提供了GB/T 28768-2012^[4]^的方法用于测定车用汽油组成,该方法采用一系列不同类型色谱柱对汽油中的组分按照碳数和类型分离检测,但无法得到详细的单体烃组成信息。行业标准SH/T 0714-2002^[5]^采用50 m长的高分辨弹性石英毛细管色谱柱测定汽油馏分单体烃组成,该方法已获得了广泛应用,但用于车用汽油单体烃组成测定时,会存在添加的含氧化合物或非常规添加组分与烃类组分间的相互干扰。ASTM D6729-20^[6]^和ASTM D6730-21^[7]^的方法均采用100 m长的毛细管色谱柱测定汽油单体烃,可以使部分含氧化合物与烃类实现分离,但仍存在部分含氧化合物与烃类的干扰及烷基化汽油组分与芳烃的重叠,使方法应用于成品汽油分析时受到一定限制。近年来,也有采用全二维色谱技术用于汽油组成分析的应用研究,在汽油组成及芳烃分析^[8⇓⇓⇓-12]^、含氧化合物及非常规添加组分分析^[13⇓-15]^等方面取得了较好的效果,但仍无法直接用于车用汽油详细单体烃组成分析。

气相色谱中心切割(Deans switch)技术采用了微板流控技术,与色谱柱的连接几乎无死体积,通过电子流量精确控制,可以实现毛细管色谱柱之间目标组分的精准切割,在汽油中的微量含氧化合物测定、非常规添加组分测定方面获得了成功的应用^[16⇓⇓⇓-20]^,并建立了相应的标准方法^[3,21]^。本文将中心切割技术与高分辨毛细管色谱分离相结合,开发了采用双通道三色谱柱和三检测器测定车用汽油详细组成的方法,在一个通道采用PONA柱实现常规单体烃测定的基础上,另一个通道通过中心切割的方式,将PONA柱上难分离的含氧化合物、苯胺类化合物和烃类组分切换至DM-624色谱柱上进一步分离,通过双通道联合计算,获得车用汽油的详细单体烃和含氧化合物及苯胺类化合物的组成结果。

## 1 实验部分

### 1.1 仪器和试剂

Agilent 8890气相色谱仪,配有2个分流/不分流进样口、3个火焰离子化检测器(FID)、两个自动进样器和一个Deans switch中心切割配件(美国安捷伦公司)。

甲醇、乙醇、异丙醇、正丙醇、异丁醇、仲丁醇、叔丁醇、正丁醇、MTBE、ETBE、DIPE、TAME、甲缩醛、乙酸乙酯(EA)、SBA、碳酸二甲酯(DMC)、苯胺、*N*-甲基苯胺、邻-甲基苯胺和间-甲基苯胺、正庚烷、正壬烷均为分析纯。

催化裂化汽油和烷基化汽油来自中石化燕山石化分公司;国Ⅴ-92、国Ⅴ-95、国Ⅵ-92和国Ⅵ-95车用汽油、E-95乙醇汽油为市售。

### 1.2 分析系统

色谱分析系统连接示意图见图1。通道1采用常规的PONA色谱柱,连接分流进样口和FID,用于测定常规烃类详细单体烃组成;通道2由两根色谱柱组成双柱系统,第1根为PONA色谱柱,第2根采用30 m长的弱极性DM-624色谱柱,两根色谱柱通过Deans switch组件相连接。两个通道的进样采用了双自动进样器同时进样来实现。启动运行程序后,通道1按照常规的单体烃运行温度程序来进行,实现常规单体烃色谱峰分离,并进入第1个FID进行检测;通道2的样品注入进样口后,按照预先设定的中心切割时间表,在特定的时间将在PONA色谱柱上无法分离的含氧化合物及非常规添加组分等切割至第2根DM-624色谱柱实现进一步的分离,并进入第2个FID进行检测。第三个检测器可以采用火焰离子化检测器或热导检测器(TCD),用于监测通道2中PONA色谱柱的分离情况。通道2的PONA柱通过一段与DM-624色谱柱阻尼相当的阻尼柱与第三检测器相连接。在确定阀切换时间表时,首先不进行阀切换,让所有组分全部通过阻尼柱连接的FID检测,这样得到的组分保留时间就等于组分离开PONA柱的时间,根据色谱峰起始和回到基线的时间确定对某个色谱峰进行中心切割的起始和结束时间,实现组分的精准切割。

**图1:**
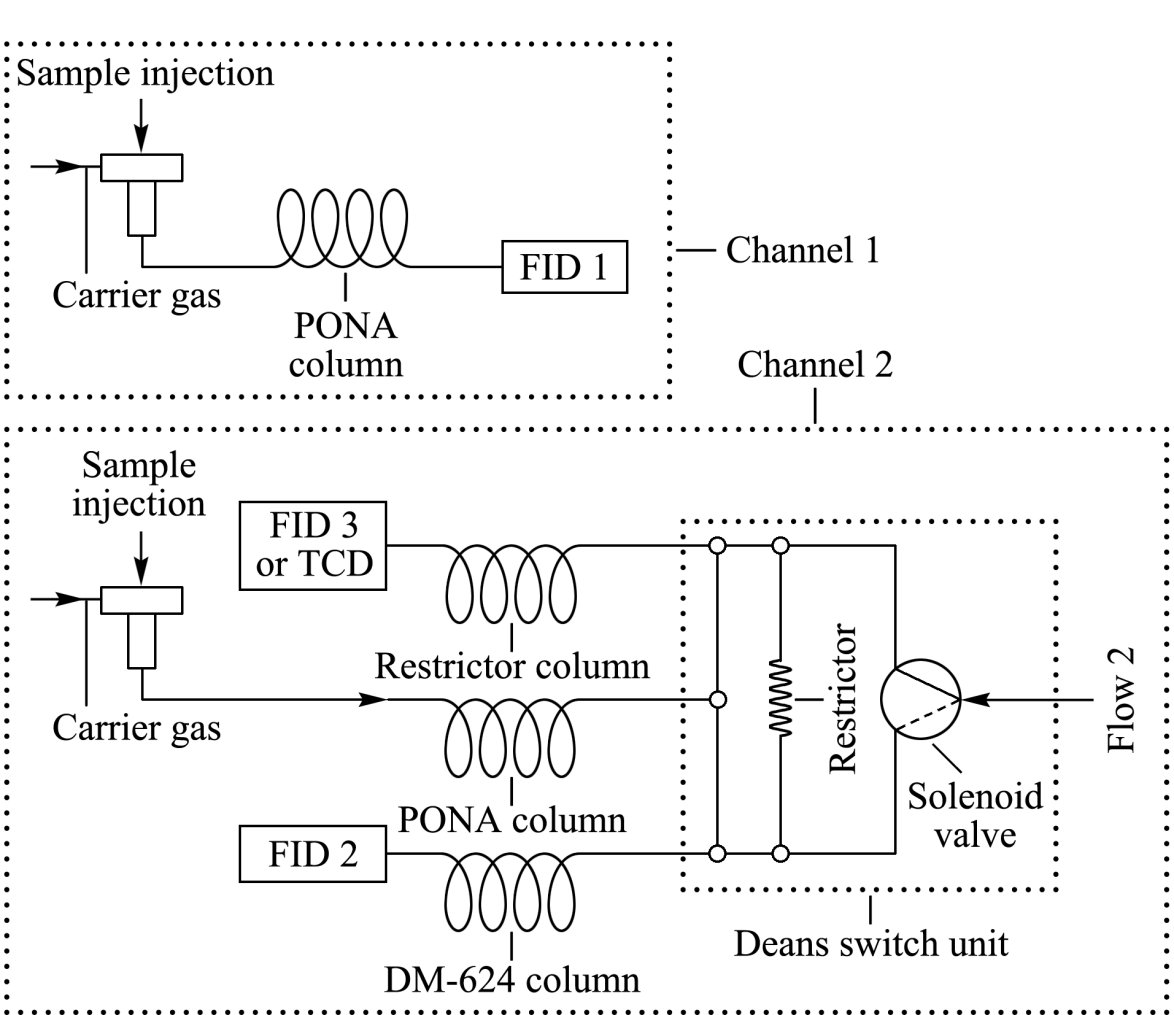
双通道三色谱柱系统测定成品汽油详细组成的气路连接示意图

根据两个通道FID得到的原始色谱峰积分结果,经两个通道的联合运算,可以得到成品车用汽油的详细组成结果。方法测定过程示意图见图2。

**图2:**
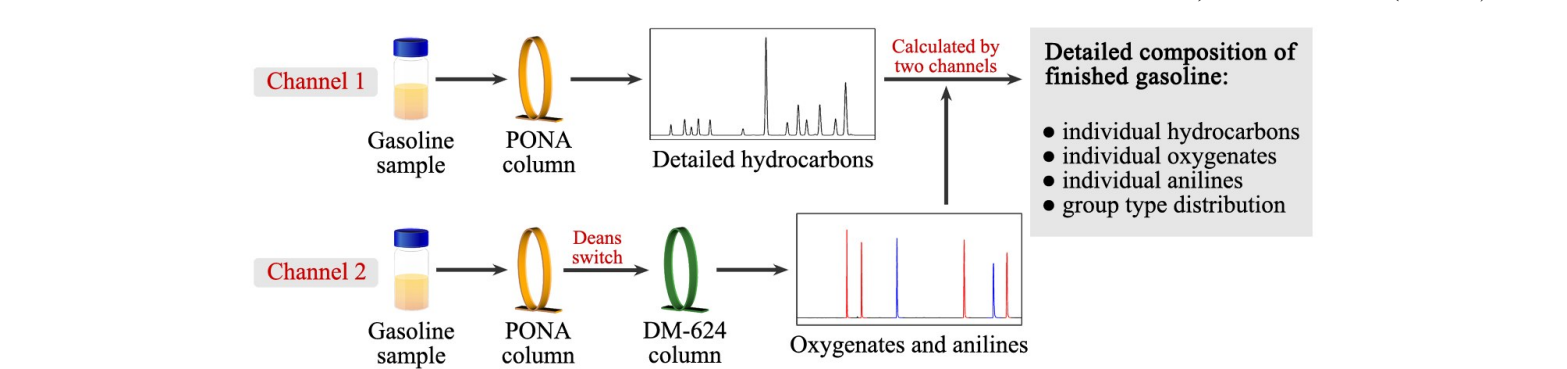
成品汽油详细组成测定流程示意图

### 1.3 色谱条件

色谱柱:PONA柱(50 m×0.20 mm×0.5 μm)(美国安捷伦公司); DM-624(30 m×250 μm×1.4 μm)(北京迪科马科技有限公司)。进样口温度:250 ℃;检测器温度:250 ℃;柱温条件:35 ℃保持15 min,以2 ℃/min的升温速率升至180 ℃;载气为N_2_,恒压模式,前进样口压力181.33 kPa,后进样口压力98.60 kPa,第二路压力控制模块(PCM)压力126.59 kPa;分流比100∶1,进样量0.2 μL;检测器为FID,氢气流量30 mL/min,空气流量350 mL/min,尾吹气(N_2_)流量25 mL/min。

### 1.4 非烃组分混合溶液制备

在100 mL容量瓶中,分别取2 mL甲醇、乙醇、正丙醇、异丙醇、正丁醇、异丁醇、叔丁醇、MTBE、TAME、DIPE、ETBE、甲缩醛、SBA、EA、DMC、苯胺、*N*-甲基苯胺、邻-甲基苯胺和间-甲基苯胺,用正壬烷稀释至刻度,混合均匀,得到含氧化合物和非常规添加组分体积分数均为2%的非烃组分混合溶液。

## 2 结果和讨论

### 2.1 非烃组分和烃类组分在PONA色谱柱上的分离

为考察含氧化合物和非常规添加组分与烃类组分在PONA色谱柱上的分离情况,取0.2 μL前述非烃组分混合溶液注入通道1并采用1.3节色谱条件进行测定。FCC汽油是车用汽油的基础调合组分汽油,由于含有大量烯烃组分,在所有调合组分汽油中组成最为复杂。在相同色谱条件下将FCC汽油样品注入通道1进行测定。将这两个样品的色谱图叠放,见图3,其中蓝色谱图代表FCC汽油的色谱图,均为烃类组分的色谱峰,红色谱图为含氧化合物和苯胺类化合物混合溶液的色谱图。由图3可以看到,部分含氧化合物与烃类组分存在共流出,无法直接定量。以30 min前的放大谱图为例,对于乙醇、正丙醇、异丙醇等组分,因其色谱峰(红色)与烃类色谱峰(蓝色)不存在重叠,可以直接在通道1的PONA色谱柱上进行定性定量;对于甲醇、MTBE等组分,因其色谱峰(红色)与烃类色谱峰(蓝色)共流出,以及甲缩醛和叔丁醇、乙酸乙酯和碳酸二甲酯等存在共流出的组分,需要通过设定中心切割的方式,将它们切割至DM-624色谱柱上实现进一步的分离。

**图3:**
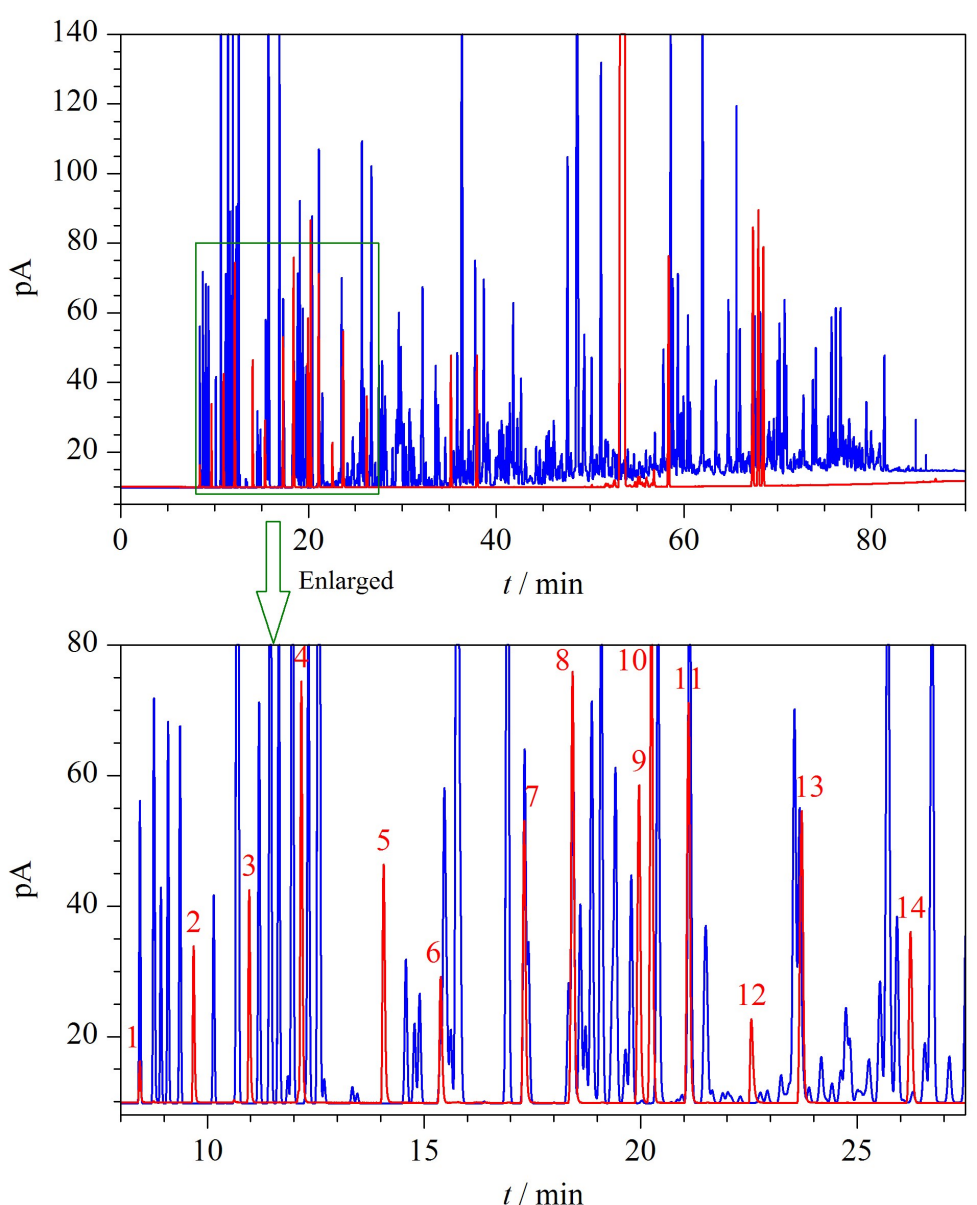
FCC汽油、含氧化合物和苯胺类化合物在PONA色谱柱上的色谱图

### 2.2 色谱柱系统的选择

为使含氧化合物及非常规添加组分与烃类组分从PONA色谱上的共流出峰切割至第2根色谱柱上获得较好的分离效果,比较了DB-225MS(60 m×0.25 mm×0.25 μm)、DB-17MS(60 m×0.25 mm×0.25 μm)、DB-1701(60 m×0.25 mm×0.25 μm)和DB-624(60 m×0.25 mm×1.4 μm)等不同类型固定相的色谱柱(均购自美国安捷伦公司)对常规醇醚含氧化合物及非常规添加组分和烃类的分离情况。几种色谱柱对苯胺类化合物分离效果基本相当。由于苯胺类化合物在色谱柱上出峰时间较长,截取了几种色谱柱对含氧化物的分离谱图(见图4)。

**图4:**
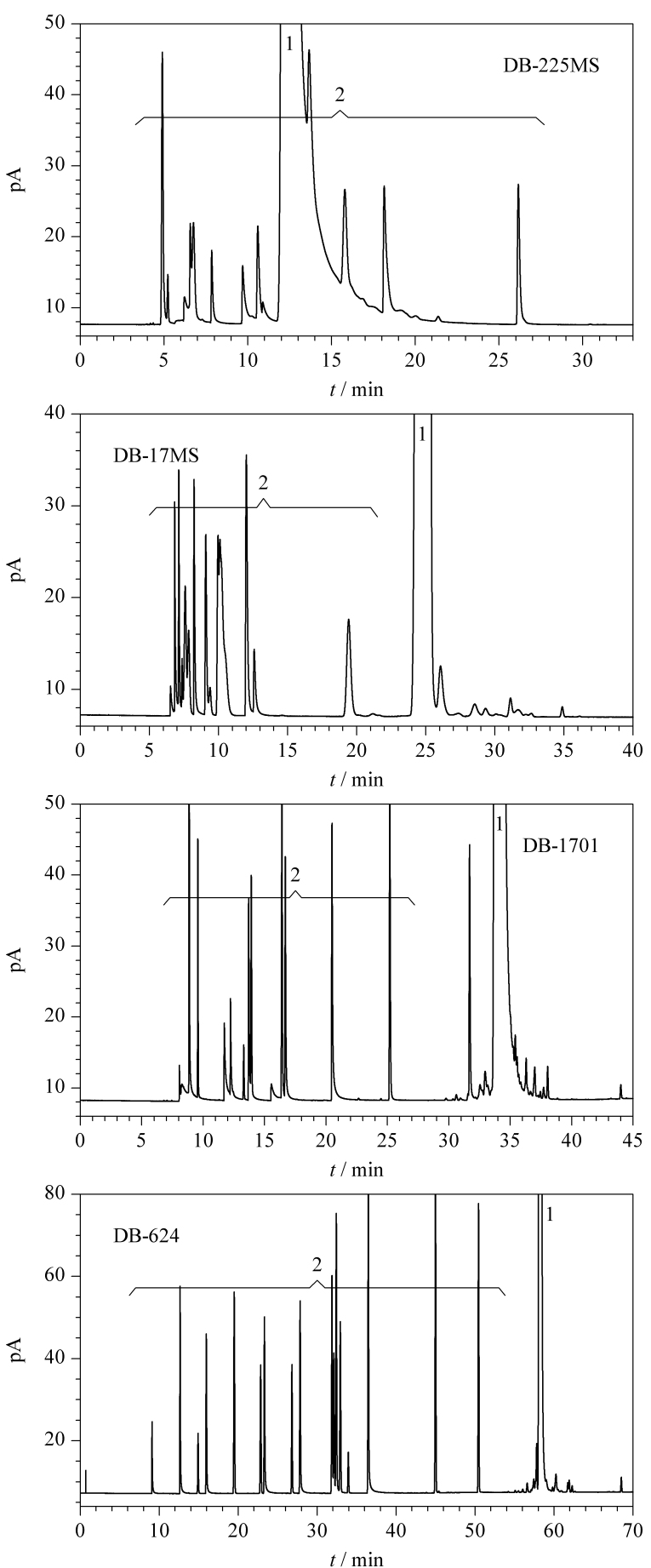
含氧化合物在不同类型色谱柱上的分离谱图

结果表明,采用极性较强的色谱柱,对于芳烃有较好的分离效果,但对于弱极性的醚类如MTBE与烃类的分离存在着拉平效应,无法实现二者分离。同时由于升温程序采用了从35 ℃开始的升温过程,在较低的温度下,较强极性的色谱柱对醇类等极性化合物的保留能力较强,使得峰形展宽和拖尾,影响分离效果。当采用偏弱极性的DB-624色谱柱时,可以获得含氧化合物与烃类组分的较好分离。因此确定采用DB-624色谱柱为柱2进行目标色谱峰的分离。为比较色谱柱2的分离效果,分别考察了DB-624(60 m×0.25 mm×1.4 μm)、DM-624(50 m×0.25 mm×0.5 μm)和DM-624(30 m×0.25 mm×1.4 μm)3种同一类型固定相不同尺寸的色谱柱,比较其对含氧化合物的分离情况,综合考虑中心切割对流量的匹配要求、色谱柱的分离效果和分析时间,确定采用30 m×0.25 mm×1.4 μm的DM-624色谱柱作为色谱柱2,用于分离含氧化合物和非常规添加组分。

### 2.3 中心切割时间表的确立和组分定性

为选择合适的中心切割时间,在通道2分析非烃组分混合溶液和FCC汽油样品,将中心切割的电磁阀置于“关”的位置,让组分全部通过通道2的第1根PONA色谱柱后进入第三个检测器检测。根据通道2上各非烃组分和烃类组分在PONA色谱柱上的实际保留时间,确定了各组分的中心切割电磁阀的开关时间表,见表1。对于表1中未列出的乙醇、正丙醇、异丙醇、异丁醇、ETBE和TAME,由于在PONA色谱柱上烃类组分峰的干扰不明显(见图3),不用进行中心切割,可直接采用通道1的PONA色谱柱上的色谱峰来计算。当阀切换时间表确定后,在实际分析中,对于全部采用电子流量控制的色谱仪器,只要保证色谱条件不变,阀切换时间表即保持不变。实际应用中,通过观察第三个检测器得到的通道2中PONA柱的色谱图,可以获知阀切换时间是否合适。如果观察到色谱峰切割不完全,则需要重新分析非烃组分混合溶液,根据保留时间对阀切换时间表做出调整。

**表1:** 中心切割阀切换时间表

No.	Times for solenoid valve ON/min	Times for solenoid valve OFF/min	Target components
1	8.10	8.50	methanol
2	11.80	12.30	methylal & tert-butanol
3	15.10	15.55	MTBE
4	16.50	17.60	sec-butanol, 3-methylpentane
5	18.10	18.80	EA & DMC & DIPE
6	23.40	23.90	n-butanol
7	29.50	29.90	n-heptane
8	34.95	35.55	sec-butyl acetate
9	36.00	36.85	toluene & 2,3,3-trimethyl-
			pentane
10	58.15	58.75	aniline
11	67.05	69.00	N-methyl aniline, o-methyl
			aniline, p/m-methyl aniline

采用1.3节的色谱条件和表1的中心切割时间对非烃组分混合溶液以及FCC汽油样品进行分析,得到它们在DM-624柱上的色谱图,见图5。图中蓝色部分为FCC汽油的色谱图,红色部分为非烃组分的色谱图。由于进行了多次的阀切换操作,烃类和含氧化合物及非常规添加组分分时间段进入到DM-624色谱柱上分离。8.10 min电磁阀打开至8.50 min电磁阀关闭,甲醇和异丁烷的共流出峰从PONA色谱柱流出后被切割至DM-624柱上。图5中甲醇峰和FCC汽油中异丁烷的峰保留时间差异明显,可以获得很好的分离;阀切换时间11.80~12.30 min对应图中13~17 min的色谱峰,除蓝色的烃类组分与含氧化合物获得很好的分离外,在PONA色谱柱上无法分离的甲缩醛和叔丁醇也获得了很好的分离;15.10~15.55 min阀切换时间段对应保留时间18~19 min之间的峰,MTBE与烃类色谱峰(峰16)获得了很好分离。由于在PONA柱上MTBE与2,3-二甲基丁烷和环戊烷的色谱峰都非常接近,为保证MTBE切割完全,设定的阀切换时间段宽于MTBE的色谱峰宽,使2,3-二甲基丁烷和环戊烷一起切割至DM-624色谱柱上并成为合峰(峰16)。由于2,3-二甲基丁烷和环戊烷在PONA柱上能够分离,因此只需获得MTBE的准确峰面积用于后续的校正计算。同理其他时间段都获得了目标组分与烃类的很好分离。由于3-甲基戊烷在通道1的PONA柱上是一个独立的色谱峰,因此选择其作为参考峰,将其切割至DM-624色谱柱上(峰17),用于根据它的色谱峰面积进行后续的校正计算。

**图5:**
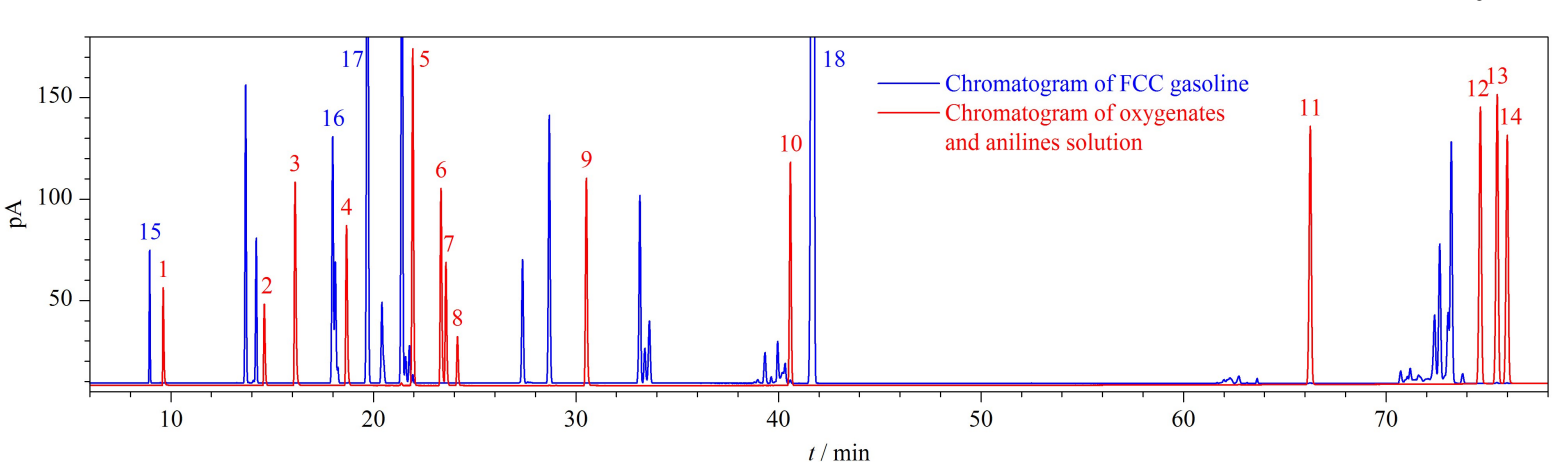
DM-624色谱柱上组分的色谱图

对于国Ⅵ成品汽油,目前广泛调入了烷基化汽油。2,3,3-三甲基戊烷是烷基化汽油中的一个主要组分,它在PONA色谱柱上与甲苯无法分离,即使采用100 m长的非极性色谱柱,也很难获得较好的分离效果,影响甲苯组分定量,给样品芳烃含量测定带来偏差。表1中给出了将甲苯和2,3,3-三甲基戊烷的共流出峰切割至DM-624色谱柱上的阀切换时间。按照1.3节色谱条件分别分析烷基化汽油和一个未调入烷基化汽油的国Ⅴ-92号车用汽油,并在36.00 min打开电磁阀、在36.85 min关闭阀,得到在DM-624色谱柱上的色谱图,见图6。可以看出,烷基化汽油所含2,3,3-三甲基戊烷和国Ⅴ-92号车用汽油中甲苯色谱峰的保留时间相差1 min,二者获得了很好的分离。

**图6:**
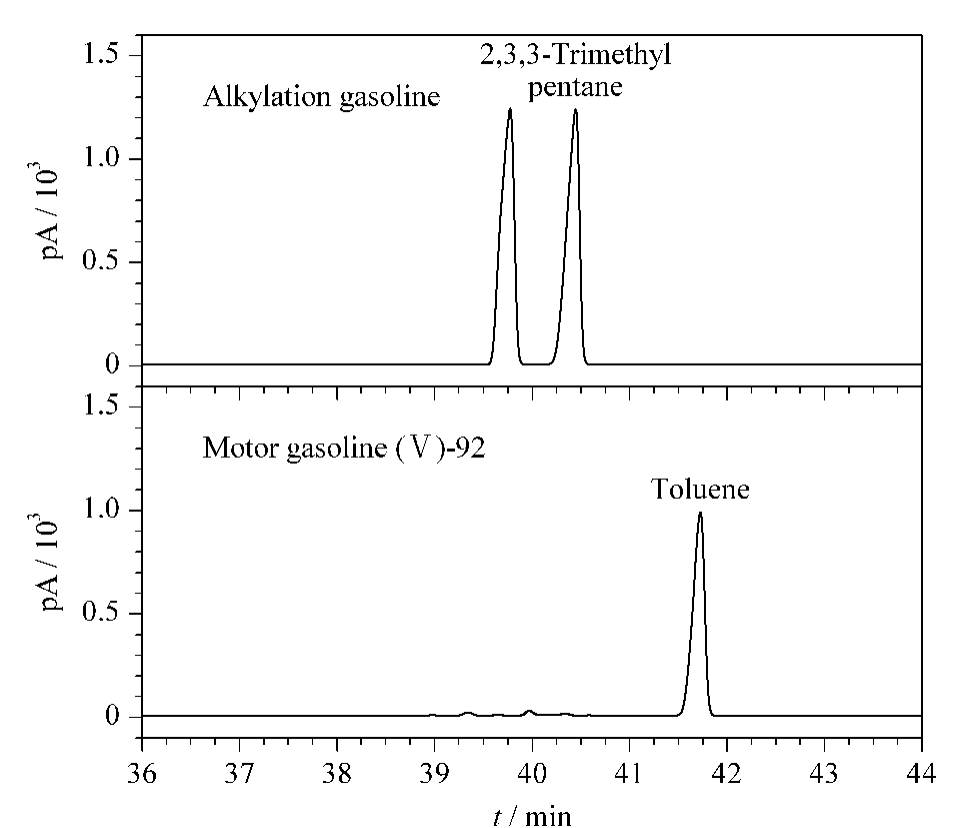
2,3,3-三甲基戊烷和甲苯在DM-624柱上的分离谱图

### 2.4 定量方法建立

定量通过通道间的校正归一化法来进行。假设在通道1的PONA色谱柱上有一由组分*i*和*k*共流出组成的色谱峰*t*,经通道2的精准切割后进入DM-624色谱柱上获得了组分*i*和*k*的分离。由于通道1和2分别有独立的进样器和进样口,无法保证实际进样量完全一致,因此,为了根据组分*i*在DM-624色谱柱上峰面积计算其在通道1 PONA柱上的峰面积,选择车用汽油中稳定存在且独立的色谱峰3-甲基戊烷作为通道间色谱峰面积换算的内标色谱峰,并按照式(1)计算*i*组分在通道1 PONA柱上的峰面积:


(1) $$A_{i,\text{ PONA }}=\frac{A_{s,PONA}}{A_{s,624}}\times A_{i,624}\times F_{i}$$

式中,*A_i_*_,PONA_为组分*i*在通道1的PONA色谱柱上的峰面积;*A*_s,PONA_、*A*_s,624_为3-甲基戊烷在通道1的PONA柱、通道2的DM-624柱上的峰面积;*A_i_*_,624_为组分*i*在DM-624柱上的峰面积;*F_i_*为通道间的面积校正系数。

通道间面积校正系数*F_i_*按照式(2)计算:


(2) $$F_{i}=\frac{f_{i,624/3mc5}}{f_{i,PONA/3mc5}}=\frac{f_{i,624/nc7}}{f_{i,PONA/nc7}}\times \frac{f_{3mc5,PONA/nc7}}{f_{3mc5,624/nc7}}$$

其中*f_i_*_,624/3mc5_、*f_i_*_,PONA/3mc5_、*f_i_*_,624/nc7_、*f_i_*_,PONA/nc7_为组分分别在通道2的DM-624柱和通道1的PONA柱上测定得到的相对于3-甲基戊烷和相对于正庚烷的相对质量响应因子;*f*_3mc5,PONA/nc7_、*f*_3mc5,624/nc7_为3-甲基戊烷分别在通道1的PONA色谱柱和通道2的DM-624色谱柱上测得的相对于正庚烷的相对质量响应因子。

当检测器采用FID时,各组分通道间校正系数都应接近于1。

根据式(1)计算得到的*A_i_*_,PONA_以及共流出峰*t*的峰面积(*A_t_*_,PONA_),由式(3)拆分计算组分*k*在通道1 PONA色谱柱上的色谱峰面积(*A_k_*_,PONA_):


(3) *A_k_*_,PONA_=*A_t_*_,PONA_-*A_i_*_,PONA_

计算出通道1 PONA上所有需要拆分的色谱峰面积后,根据所有组分对应的色谱峰面积*A_i_*_,PONA_和相对于正庚烷的相对质量响应因子*f_i_*_,PONA/nc7_,计算各色谱峰对应的组分含量(*ω_i_*_,PONA_):


(4) $$\omega_{i,PONA}=\frac{A_{i,PONA}\times f_{i,PONA/nc7}}{\sum \left(A_{i,PONA}\times f_{i,PONA/nc7}\right)}\times 100\%$$

由于数据处理时需对分离得到的300多个组分进行逐一定性,定量需通过两个通道的色谱峰面积的联合计算,因此无法在常规的色谱工作站中直接完成。为此,我们在前期开发的汽油单体烃分析方法软件的基础上^[22]^,开发了专门用于该方法的计算软件,建立了定性数据库,通过计算样品中色谱峰的色谱保留指数并与数据库比对进行定性,定量采用校正归一化,非烃组分的校正因子采用实测值,烃类组分校正因子选用文献[7]给出的数值。

### 2.5 测定的重复性和准确性考察

#### 2.5.1 中心切割的重复性考察

所建立的方法在一次运行中需根据不同色谱峰的保留时间进行多次切割,因此在实际操作中要求色谱峰的保留时间有很好的重复性。图7分别为随机选取的2021年7月、2022年7月、2023年12月3个92号汽油在通道2的PONA色谱柱上部分组分流出的色谱图,期间完成了上千次的分析测定。由色谱图可知,本工作采用的带有载气电子流量控制的色谱系统以及商业化的PONA色谱柱,保证了同一组分色谱峰保留时间在经过上千次运行后仍保持很好的重复性,同一组分保留时间的绝对偏差一般为0.01~0.03 min,确定的阀切换时间表不需调整,可以满足中心切割对色谱峰精准切割的要求。

**图7:**
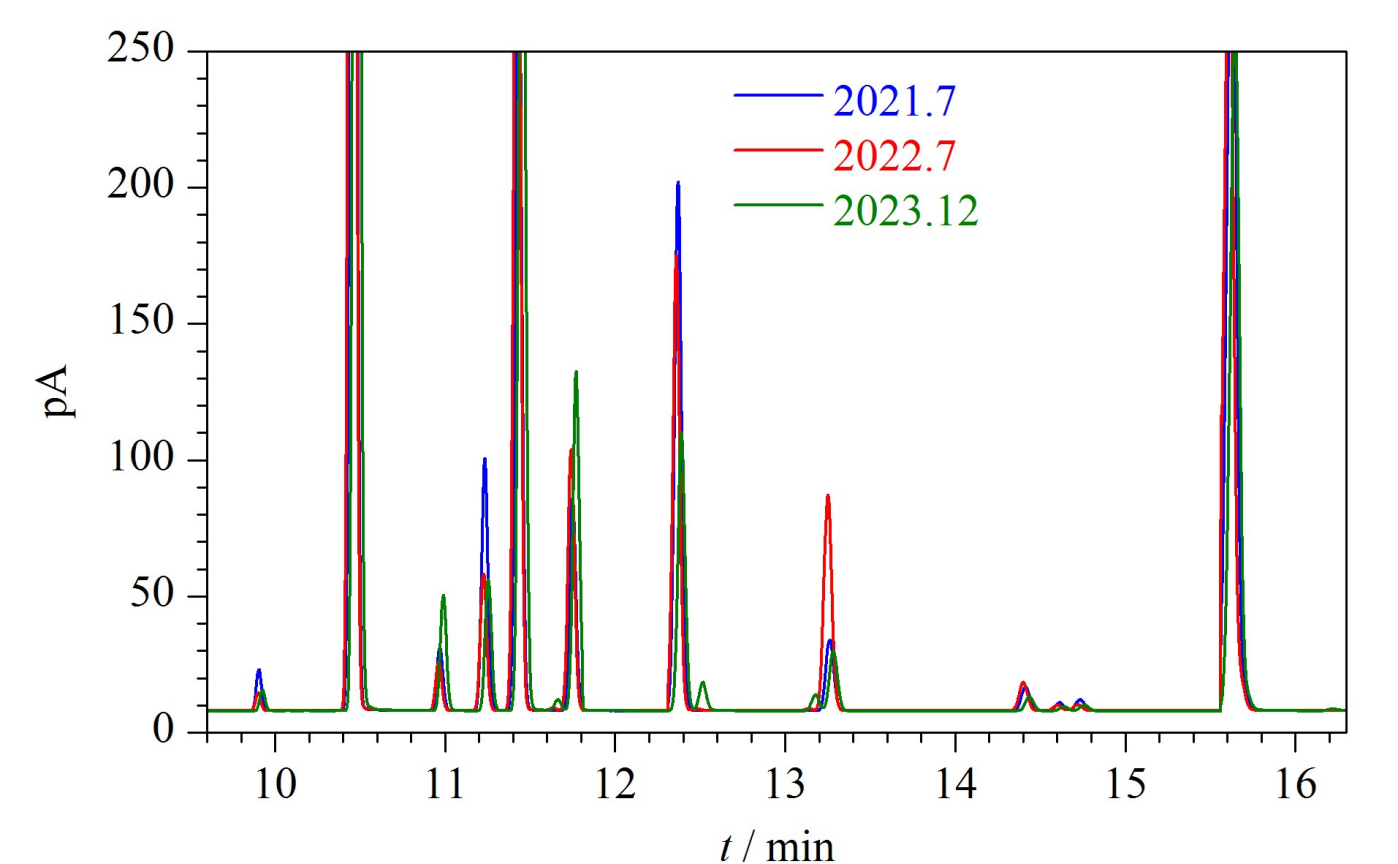
组分在通道2 PONA色谱柱上2年间保留时间的重复性

#### 2.5.2 测定结果的重复性和回收率

取一市售国Ⅴ-92号成品汽油,加入质量分数各约3%的醇醚含氧化合物和甲缩醛、苯胺等非常规添加组分,配制成两个含有含氧化合物和非常规添加组分的试样,连续测定6次,计算测定平均值、RSD和回收率,见表2。结果显示,各目标组分测定结果的RSD为0.2%~5.1%,回收率为90.1%~118.2%。

**表2:** 含氧化合物和苯胺类化合物在国Ⅴ-92号成品汽油中测定的重复性和加标回收率(*n*=6)

Sample No.	Component	Background/%^*^	Spiked/%^*^	Found/%^*^	RSD/%	Recovery/%
1	methanol	0.02	2.85	2.85	1.0	99.3
	methylal	-	3.33	3.17	0.3	95.2
	tert-butanol	0.02	2.79	2.87	0.6	102.0
	MTBE	3.29	2.52	5.77	0.5	98.3
	DIPE	-	2.94	3.07	0.5	104.1
	sec-butanol	-	2.87	2.93	0.6	102.1
	ethyl acetate	-	2.89	3.01	0.6	104.3
	DMC	-	3.17	3.32	0.6	104.5
	n-butanol	-	3.50	3.58	0.7	102.2
	SBA	-	2.96	3.07	0.7	103.7
2	aniline	-	2.57	2.59	5.1	100.5
	N-methyl aniline	-	2.57	2.51	1.3	97.9
	o-methyl aniline	-	2.79	2.74	1.4	98.2
	m-methyl aniline	-	2.62	2.55	1.3	97.2
	ethanol	-	2.72	2.79	1.3	102.6
	iso-propanol	-	2.66	2.80	2.3	105.0
	n-propanol	-	2.97	3.13	2.5	105.5
	iso-butanol	-	2.70	3.19	3.2	118.2
	ETBE	-	2.62	2.37	1.1	90.1
	TAME	-	2.69	2.73	0.2	101.7

* Mass fraction.

另取典型乙醇汽油样品连续测定6次,并按照碳数族组成分类计算各类型组分按照碳数和类型分布的质量分数。结果显示,各碳数族组成质量分数测定结果的RSD除含量很低的正十二烷(0.1%)和C_10_环烷烃(0.19%)分别为5.2%和3.3%外,均小于3%,可以满足日常测定的要求。

### 2.6 实际样品测定

选择不同来源的几个典型国Ⅴ、国Ⅵ的92号和95号汽油和乙醇汽油,采用建立的方法进行了测定,根据得到的详细单体烃组成结果计算族组成并根据组分和样品的密度换算为体积分数,与标准方法结果对比见表3。某一典型的国Ⅵ汽油的色谱图见图8。表3结果显示,本研究建立的方法与标准方法所得结果一致,可以满足一般日常分析测定的需要。同时本研究还可以得到成品调合汽油的详细单体烃组成信息,可以为汽油的精准调合和品牌汽油的研究提供基础数据。

**表3:** 实际成品汽油采用本文方法与标准方法测定的结果

Sample type	Method^1)^	Benzene/%^2)^	Aromatics/%^2)^	Olefins/%^2)^	Total oxygen content/%^3)^	Ethanol/%^2)^
Motor gasoline (Ⅴ)-92	this method	0.80	28.2	15.6	1.2	-
	standard method	0.75	28.4	13.3	1.1	-
Motor gasoline (Ⅴ)-95	this method	0.43	39.0	7.7	2.1	-
	standard method	0.41	39.9	6.2	2.0	-
Motor gasoline (Ⅵ)-92	this method	0.59	31.3	8.1	1.6	-
	standard method	0.60	33.3	5.9	1.5	-
Motor gasoline (Ⅵ)-95	this method	0.52	31.1	7.8	2.5	-
	standard method	0.50	32.2	6.0	2.2	-
Ethanol gasoline (Ⅴ)-	this method	0.73	35.2	13.1	-	11.1
E95	standard method	0.69	35.6	12.5	-	10.3

1) Standard method for benzene is SH/T 0693-2000^[23]^, for ethanol and total oxygen content is NB/SH/T 0663-2014, and for olefins and aromatic is GB/T 30519-2016. 2) Volume fraction. 3) Mass fraction.

**图8:**
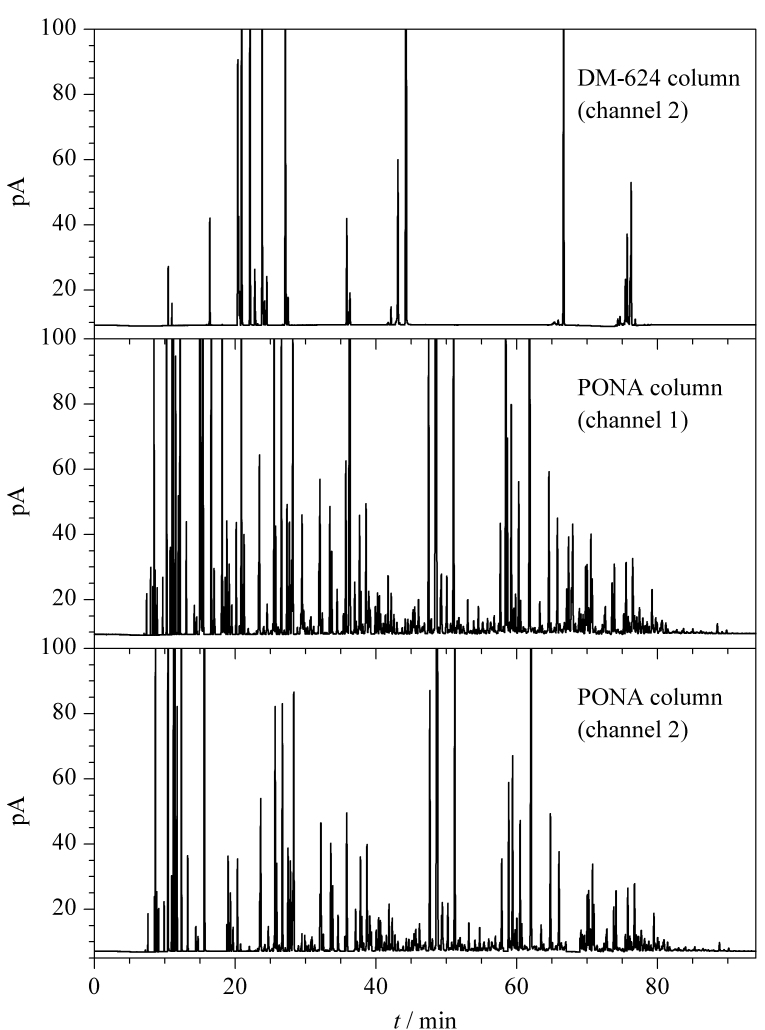
国Ⅵ-92汽油的典型色谱图

### 2.7 探讨——采用热导检测器作为第三检测器

方法中用到的第三个检测器仅用于方法建立之初确定合适的阀切换时间表和日常测定时监控中心切割运行是否正常,并不直接参加组分的定量计算,而一些常规色谱仪并不同时配置3个FID。许多色谱仪允许配置2个FID和1个TCD。为此,在方法开发过程中也考察了采用TCD作为第三个检测器的情形。当对色谱条件进行一定的调整和优化后,同样可以满足测定的要求,但需要对分析过程做以下调整和优化:(1)为保证TCD测定的灵敏度,以方便确定合适的阀切换时间表,需要将通道2的载气改为氢气,同时采用较大的进样量,实验中采用分流比150∶1和1 μL进样量即可满足检测要求。(2)由于采用氢气作为载气,载气最佳线速度要高于氮气,整体分析速度比采用氮气载气时更快,因此,对应的阀切换时间表均要做相应调整。(3)由于采用了较大的进样量,对于宽馏分的样品,不同组分存在一定的分流差异,尽管DM-624色谱柱连接的检测器为FID,但实际测定得到的部分组分对于正庚烷的相对响应因子与通过通道1 PONA色谱柱测定得到的结果存在一定的差异,即通道间的校正系数不为1。通过准确配制含有不同含量的含氧化合物、非常规添加组分和正庚烷的正壬烷溶液,可测定得到组分相对于正庚烷的相对质量响应因子。对于3-甲基戊烷响应因子的测定,进样分析1个不含烯烃的石脑油样品,并在设定的切割时间将3-甲基戊烷和正庚烷一起切至DM-624色谱柱上检测。根据通道1的PONA色谱柱和通道2的DM-624色谱柱上分别得到的3-甲基戊烷与正庚烷的色谱峰面积的比值,可以计算出两个通道间3-甲基戊烷相对于正庚烷的相对质量响应因子的比值(*f*_3mc5,PONA/nc7_/*f*_3mc5,624/nc7_),根据配制的已知浓度的组分和正庚烷的混合溶液测定得到组分在两个通道上相对于正庚烷的相对质量响应因子,利用式(2)求得通道间的校正系数*F_i_*。

由于采用TCD检测灵敏度较低、定量校正相对复杂,因此有条件的情况下尽量采用3个FID的配置更为方便可靠。

## 3 结论

建立了双通道三柱结合中心切割技术测定成品车用汽油详细单体烃组分的系统和方法,通道1保留了常规汽油单体烃分析的条件和分离效果。通道2中通过中心切割技术,将车用汽油中在PONA色谱柱上与烃类组分难分离的醇醚类含氧化合物和需要检测的禁止人为添加的甲缩醛、苯胺等非常规添加组分及部分关键难分离烃类混合组分切割至DM-624色谱柱上实现了分离,根据DM-624色谱柱上分离得到的难分离组分的峰面积和通道间的定量校正系数,推算出这些难分离组分在通道1的PONA色谱柱上的共流出峰中所占的色谱峰面积,从而实现共流出色谱峰的拆分。根据通道1的PONA柱上所有色谱峰的峰面积和定性结果,采用校正归一的方法可以得到车用汽油中含氧化合物、非常规添加组分和单体烃质量分数,并进一步计算得到样品的碳数族组成数据,消除了含氧化合物及非常规添加组分与烃类组分间的干扰问题,实现了成品车用汽油详细组成的分析测定。该法有较好的重复性和回收率,对实际不同标号车用汽油测定得到的不同类型组分的测定结果与标准方法GB/T 30519-2016、NB/SH/T 0663-2014及SH/T 0693-2000的测定结果有很好的一致性。对于添加了甲缩醛等某些非常规添加组分的样品,也可以得到这些组分的含量。该方法通过一次分析可以得到常规分析需要3~4个方法的分析数据,同时可以获得车用汽油的详细单体烃组成信息,可为汽油的精准调合及质量控制提供支持,有很好的应用前景。
